# Nigerian foodstuffs with prostate cancer chemopreventive polyphenols

**DOI:** 10.1186/1750-9378-6-S2-S9

**Published:** 2011-09-23

**Authors:** Sunday Eneojo Atawodi

**Affiliations:** 1 Biochemistry Department, Ahmadu Bello University, Zaria, Nigeria

## Abstract

Dietary polyphenols are antioxidants that can scavenge biological free radicals, and chemoprevent diseases with biological oxidation as their main etiological factor. In this paper, we review our laboratory data vis-ὰ-vis available literature on prostate cancer chemopreventive substances in Nigerian foodstuffs. *Dacryodes edulis* fruit, *Moringa oleifera* and *Syzygium aromaticum* contained prostate active polyphenols like ellagic acid, gallate, methylgallate, catechol, kaempferol quercetin and their derivatives. Also *Canarium schweinfurthii* Engl oil contained ten phenolic compounds and lignans, namely; catechol, p-hydroxybenzaldehyde, dihydroxyphenylacetic acid, tyrosol, p-hydroxybenzoic acid, dihydroxybenzoic acid, vanillic acid, phloretic acid, pinoresinol, secoisolariciresinol. In addition, tomatoes (*Lycopersicon esculentum* Mill) which contains the powerful antioxidant and anti-prostate cancer agent, lycopene; cabbage (*Brassica oleracea*) containing indole-3-carbinol; citrus fruits containing pectin; Soursop (*Annona muricata*) containing annonaceous acetogenins; soya beans (*Glycine max*) containing isoflavones; chilli pepper (*Capsicum annuum*) containing capsaicin, and green tea (*Camellia sinensis*) containing (-) epigallocatechin gallate (EGCG), (-) epicatechin, (-) epicatechin-3-gallate and (-) epigallocatechin -3-gallate which are widely reported to posses prostate cancer chemopreventive compounds are also grown in Nigeria and other African countries. Thus, the high incidence of prostate cancer among males of African extraction can be dramatically reduced, and the age of onset drastically increased, if the population at risk consumes the right kinds of foods in the right proportion, beginning early in life, especially as prostate cancer has a latency period of about 50 years.

## Introduction

Cancer is a debilitating disease that has afflicted a good proportion of the world population in all generations. According to recent estimates by the WHO [1], annual cancer incidence in sub-Saharan Africa is 551,200 with a mortality of 421,000. Out of these cases, prostate cancer constitute 24 % , and is the fifth most common of all cancers. It is the cancer with the highest incidence, and consequently, responsible for the highest mortality rate of all cancers among black males in sub-saharan Africa. With this unenviable status in the black race, prostate cancer deserve every possible attention with respect to control, prevention and therapy.

According to Adhami *et al*[2], prostate cancer is an ideal candidate disease for chemoprevention because it is typically diagnosed in men ages >50 years and has a high latency period, and hence, even a slight delay in the progression of this disease by chemopreventive intervention could result in a substantial reduction in the incidence of the disease and, more importantly, improve the quality of life of the patients. Epidemiological and laboratory evidence also indicate that the differences in incidence of cancers, in general, and prostate cancer in particular [3], may be associated with the presence of certain polyphenols, especially flavonoids in the diets of these populations [4]. In this paper, we review our research data vis-a-vis published information on cancer chemopreventive polyphenols in Nigerian foods with particular reference to those that have been reported to be active against prostate cancer.

## Materials and methods

### Chemicals and reagents

Chemicals and reagents are of HPLC grade obtained from either Merck (Darmstadt, Germany). Serva (Heidelberg, Germany), Aldrich Chemie (Steinheim, Germany) or Fluka (Buchs, Switzerland). Standard phenolic compounds were obtained from laboratory stock maintained at German Cancer Research Centre, Heidelberg, Germany. All reagent solutions were made in double-distilled water.

### Sample and sample extraction

Sun-dried food samples were acquired from retail outlet in Zaria, Kaduna State, Nigeria in July, 2004. The samples were prepared, stored and extracted with methanol on Soxhlex apparatus following prior extraction with hexane as previously reported [5,6]. Extraction of the *Canarium schweinfurthii* Engl fruit oil was achieved by solvent-solvent extraction [7].

### Analytical high performance liquid chromatography and liquid chromatography-electrospray ionization mass spectrometry

This was conducted on a Hewlett-Packard (Agilent Technologies, Waldbronn, Germany) model 10980 liquid chromatograph (Latex, Eppelheim, Germany). Chromatographic separation was conducted using a C-18 reverse-phase (particle size, 5 µm) column (25 cm × 2mm i.d.; Latex). For separation of individual compounds in the extract, 2% acetic acid in water (solvent A) and methanol (solvent B) were used as mobile phase when 20 µL of the extract was injected. The solvent gradient consisted of 95% A for 2 minutes, 75% A in 8 minutes, 60% A in 10 minutes, 50% A in 10 minutes, and 0% A until completion of the run at 45 minutes. The flow rate of the mobile phase was maintained at 1 mL/minute (and 0.5mL/Minute for liquid chromatography-electrospray ionization mass spectrometry), and phenolic compounds in the eluate were detected with an ultraviolet dual-array detector (HP1040M, Hewlett-Packard) set at 278 and 340 nm for analytical HPLC. For liquid chromatography- electrospray ionization mass spectrometry, the analyses were conducted in the negative ion mode under the following conditions: dry gas (nitrogen) flow rate, 10 L/minute; nebulizer pressure = 30 psi, drying gas temperature = 350°C; capillary voltage = 2,500 V; fragmenter voltage = 100 V; mass range = 50–3,000 Da.2. Instrument control and data handling were by means of HP Chemstation (Hewlett-Packard) operating in the Microsoft Windows (Redmond, WA, USA) software environment. The amount of phenolic compounds in the extracts was estimated by the external standard method [5,6].

### Antioxidant activity and radical scavenging activity

The hypoxanthine/xanthine oxidase assay system the 2-deoxyguanosine assay models were adopted to evaluate the antioxidant and radical scavenging capacity of the extracts [5-7]. The amounts of extracts producing the IC50 for these model systems were determined using the Table Curve Program (Jandel Scientific, Chicago, IL, USA), and the result expressed as µL from 5g/10 mL solution; 1 µL = 0.5mg x 0.5 (dilution factor).

### Gas chromatography-mass spectrometry analysis

Analyses were performed on a HP 5973 mass spectrometer coupled to a HP 6890 gas chromatograph. Prior to GC-MS analysis, dried methanolic extracts (1 µL) were derivatized by addition of BSTFA (100 µL) at 37°C for 30 minutes. Separation of the analytes was achieved using an HP 5MS capillary column (30 m × 0.25 mm i.d; 0.25 μm film thickness). Helium was used as the carrier gas with a linear velocity of 0.9 mL/second. The oven temperature program was as follows: initial temperature, 100°C; 100–270°C at 4°C/minute; and maintained at 270°C for 20 minute. The gas chromatograph injector temperature was maintained at 250°C; the transfer line temperature was held at 280°C. The mass spectrometer parameters for electrospray ionization mode were as follows: ion source temperature, 230°C; electron energy, 70 eV; filament current, 34.6 µA; electron multiplier voltage, 1,200 V.

## Results

Whereas the methanolic extract of the leaves of *M. oleifera* Lam. contained chlorogenic acid, rutin, quercetin glucoside, and kaempferol rhamnoglucoside, the root and stem barks contained several procyanidin derivatives. Using the xanthine oxidase model system, the extracts exhibited strong *in vitro* antioxidant activity, with IC_50_ values of 16, 30, and 38 µL for the roots, leaves, and stem bark, respectively, while potent radical scavenging capacity was observed with the 2-deoxyguanosine assay model system, with IC_50_ values of 40, 58, and 72 µL for the leaves, stem bark, and root, respectively. Methanolic extract of *Dacryodes edulis* fruit on the other hand contained gallate, methylgallate, catechol, ellagic acid, quercetin and quercetin rhamnoside, which were detectable with HPLC-MSD with no additional compound peaks found by GC-MS analysis when extract was derivatized with BSFTA. Gallate occurred in the highest amount while quercetin was present in the least quantity (0.11mg/kg). *Dacryodes edulis* fruit showed high inhibitory effect on the xanthine oxidase system (IC50= 14 µL), but the radical scavenging capacity was rather poor IC_50_ value (357 µL) when analyzed with the 2-deoxyguanosine-assay system. *Canarium schweinfurthii* Engl oil contained ten polyphenols, namely; catechol, p-hydroxybenzaldehyde, dihydroxyphenylacetic acid, tyrosol, p-hydroxybenzoic acid, dihydroxybenzoic acid, vanillic acid, phloretic acid, pinoresinol, secoisolariciresinol with IC50 of 56 µL and IC_50_=104, when assayed by xanthine oxidase and 2-deoxyguanosine method, respectively (Table 1).

## Discussion

The potent antioxidant and radical scavenging capacity observed, is a good reflection of the polyphenol composition of *Canarium schweinfurthii* Engl fruit oil, *Dacryodes edulis and Moringa oleifera* extracts (Table 1). Biological activities such as these have been credited with chemoprevention of diseases like cancer, which result, at least in part, from oxidative damage [4,8]. The difference in the polyphenol composition and antioxidant activities of different parts of *M.oleifera*, suggests that different parts of the same plant may have significantly different anticancer activity.

**Table 1:** Cancer Chemopreventive Polyphenols in some Nigerian Foodstuffs

S/No	Food (Common Names)	Foods (Botanical Names)	Polyphenol Composition
1.	African pear, African plum	*Dacryodes edulis* fruit	Gallate, methylgallate, catechol, ellagic acid, quercetin and quercetin rhamnoside

2.	Drumstick, Horse Radish Tree	*Moringa oleifera*	Chlorogenic acid, rutin, quercetin glucoside, kaempferol rhamnoglucoside, procyanidins

3.	African olive	*Canarium schweinfurthii* Engl	Catechol, p-hydroxybenzaldehyde, dihydroxyphenylacetic acid, tyrosol, p-hydroxybenzoic acid, dihydroxybenzoic acid, vanillic acid, phloretic acid, pinoresinol, secoisolariciresinol

4.	Soursop, Graviola, Brazilian pawpaw	*Annona muricata*	Annonaceous acetogenins (annomontacin, annomuricatins, annomuricins, annomutacin, annonacin, annonacinone annocatalin, annohexocin, annomonicin, annopentocin, muricins muricatetrocin, longifolicin epomuricenins, epomurinins, gigantetrocins, etc)

5.	Tomatoes	*Lycopersicon esculentum* Mill	Lycopene

6.	Cabbage	*Brassica oleracea*	Indole-3-carbinol

7.	Soya beans	(*Glycine max*)	Isoflavones (daidzein and genistein)

8.	Green tea	*Camellia sinensis*	(-) epigallocatechin gallate (EGCG), (-) epicatechin, (-) epicatechin-3-gallate and (-) epigallocatechin -3-gallate

9.	Chili pepper	*Capsicum spp*	Capsaicin

10.	*Citrus fruits*	Citrus spp	Pectins, Limonoids

Quercetin and quercetin derivatives as detected in these Nigerian foods have been reported to inhibit prostate cancer colony melanoma growth, and act as pro-apoptotic agent [9,10]. Similarly, ellagic acid, an oxidation product of gallic acid, catechol, kaempferol and its derivatives which were detected in these Nigerian foodstuffs have undergone different levels of study as possible prostate cancer chemopreventive agents, with promising results, including potent antimutagenesis, antitumour and antimetastasis properties[3,11]; effects that are also relevant to prostate cancer control and chemoprevention [3,8-11]. Besides, many of the polyphenols and lignans detected in *Canarium schweinfurthii* Engl fruit oil have been credited with, among others, prostate cancer chemoprevention activities [3,4,7,12].

In addition to these foodstuffs, other food materials widely reported to posses prostate cancer chemopreventive compounds are also grown in Nigeria (table 1). These include tomatoes which contains the powerful antioxidant and anti-prostate cancer agent, lycopene; cabbage which contains indole-3-carbinol; citrus fruits which contains pectin; Graviola or Soursop (*Annona muricata*) which contains the annonaceous acetogenins, soya beans (*Glycine max*) which contains the isoflavones, daidzein and genistein; *Capsicum* spp which contains capsaicin, and *Camellia sinensis* which contains (-) epigallocatechin gallate (EGCG), (-) epicatechin, (-) epicatechin-3-gallate and (-) epigallocatechin -3-gallate [2-4,8]. Therefore, though prostate cancer is historically more prevalent in males of African extraction, the incidence can be dramatically reduced, and the age of onset drastically increased, if the population at risk consumes the right kinds of foods in the right proportion, beginning early in life, especially as prostate cancer has a gestation period of about 50 years.

## Competing interests

The author declares that he has no competing interests.
